# Protist Predation Influences the Temperature Response of Bacterial Communities

**DOI:** 10.3389/fmicb.2022.847964

**Published:** 2022-04-07

**Authors:** Jennifer D. Rocca, Andrea Yammine, Marie Simonin, Jean P. Gibert

**Affiliations:** ^1^Department of Biology, Duke University, Durham, NC, United States; ^2^Department of Plant and Microbial Biology, North Carolina State University, Raleigh, NC, United States; ^3^University of Angers, Institut Agro, Institut National de la Recherche Agronomique, L’Institut de Recherche en Horticulture et Semences, Structure Fédérative de Recherche Qualité et Santé du Végétal, Angers, France

**Keywords:** predation, temperature, protistan bacterivory, bacterivores, aquatic microbiome, microbial respiration

## Abstract

Temperature strongly influences microbial community structure and function, in turn contributing to global carbon cycling that can fuel further warming. Recent studies suggest that biotic interactions among microbes may play an important role in determining the temperature responses of these communities. However, how predation regulates these microbiomes under future climates is still poorly understood. Here, we assess whether predation by a key global bacterial consumer—protists—influences the temperature response of the community structure and function of a freshwater microbiome. To do so, we exposed microbial communities to two cosmopolitan protist species—*Tetrahymena thermophila* and *Colpidium* sp.—at two different temperatures, in a month-long microcosm experiment. While microbial biomass and respiration increased with temperature due to community shifts, these responses changed over time and in the presence of protists. Protists influenced microbial biomass and respiration rate through direct and indirect effects on bacterial community structure, and predator presence actually reduced microbial respiration at elevated temperature. Indicator species analyses showed that these predator effects were mostly determined by phylum-specific bacterial responses to protist density and cell size. Our study supports previous findings that temperature is an important driver of microbial communities but also demonstrates that the presence of a large predator can mediate these responses to warming.

## Introduction

Understanding the biotic factors that influence global climate change is one of the most pressing goals of ecology (Van der Putten et al., 2010; Barbour and Gibert, 2021). Doing so hinges on better understanding the biotic and abiotic feedbacks that determine global carbon cycling (Jackson et al., 2017). Microbial organisms comprise 14% of all existing biomass on Earth (Bar-On et al., 2018; Flemming and Wuertz, 2019), while the entirety of the Animal Kingdom, for comparison, only represents 0.3% (Bar-On et al., 2018). Microbial decomposition is responsible for the recycling of organic matter back into food webs, thus partly subsidizing the flux of energy and matter in all ecosystems (Cordone et al., 2020; Mougi, 2020). Through respiration and decomposition of existing carbon pools, microorganisms are largely regarded as one of the most important biotic controls of the global carbon cycle (Bardgett et al., 2008; Schimel and Schaeffer, 2012; Gougoulias et al., 2014; Wang, 2018; Jansson and Hofmockel, 2020). Additionally, individual respiration rates are well known to increase with temperature among ectotherms and unicellular organisms (Gillooly et al., 2001; DeLong et al., 2017; Bond-Lamberty et al., 2018), potentially resulting in a scenario where warming begets more warming. However, temperature-mediated increases in respiration rates often plateau, or even decline over time in microbial communities (Yergeau et al., 2012; Bradford, 2013; Crowther and Bradford, 2013; Ye et al., 2020), although not always (Hartley et al., 2008; Zimmermann et al., 2012). These diverse responses thus show there is much that still needs to be understood about the processes regulating microbial respiration and function and their effects on the climate.

Temperature often mediates ecological interactions (Binzer et al., 2012; Dell et al., 2014; Uszko et al., 2017; Bernhardt et al., 2018; Garzke et al., 2019). The strength of feeding interactions, in particular, increases with temperature, as feeding rates increase among consumers to compensate for increasing metabolic demands (Gillooly et al., 2001; Dell et al., 2011). Stronger predation in turn leads to declines in prey abundance and total biomass (Gilbert et al., 2014; Garzke et al., 2019; DeLong and Lyon, 2020; Barneche et al., 2021). Because gross respiration rates are determined by standing biomass, temperature effects on predation may ultimately influence ecosystem-level processes such as community respiration rates (O’Connor et al., 2009), and thus mediate the temperature response of microbial respiration rates worldwide. In particular, bacterivory is a dominant factor leading to microbial biomass loss (Pedrós-Alió et al., 2000; Jürgens and Massana, 2008; Baltar et al., 2016), which has been proposed to influence soil respiration rates (Gao et al., 2019), and shown to affect litter decomposition in soils across temperatures (Sulman et al., 2018; Geisen et al., 2021).

Recent efforts have mapped the global distribution of nematodes—a major group of microbial predators (Nielsen et al., 2014; van den Hoogen et al., 2019, 2020). These global maps represent a step forward in clarifying the role of feeding interactions in the temperature responses of microbial communities. However, with a global biomass 200 times larger than that of nematodes (Bar-On et al., 2018), unicellular eukaryotes—collectively known as “protists”—likely play a major role in regulating microbial communities at global scales (Oliverio et al., 2020) through bacterivory (Gao et al., 2019; Erktan et al., 2020). Ciliate protists, in particular, are well-known bacterivores (Foissner and Berger, 1996), their population dynamics and feeding interactions are strongly temperature-dependent (DeLong and Lyon, 2020), and they are present in all major ecosystems (Foissner and Berger, 1996; Oliverio et al., 2020). As such, predation of microorganisms by protists can mediate the temperature response of microbial communities (Gao et al., 2019; Geisen et al., 2021), although this phenomenon has, to our knowledge, so far only been shown for one species of protist in soils (Geisen et al., 2021) and deep-sea thermal vents (Hu et al., 2021). If general, this process has the potential to strongly influence microbial respiration worldwide under warmer temperatures (Crowther et al., 2015; Geisen et al., 2018). Additionally, microbial predators respond to environmental conditions themselves in multiple ways, including changes in the traits that influence predation (Atkinson et al., 2003; Dell et al., 2011; Gibert et al., 2016), which add complexity to an already complex problem.

Recent studies addressing protist effects in microbial communities have either focused on changes in ecosystem-level processes, without examining the fine scale changes in microbiome structure at the taxa-level (Geisen et al., 2021), or have studied protist effects under extreme temperature disturbances (Thakur et al., 2021). Here, we examine the potential interactive effects of protist predation and temperature on microbial diversity, biomass, and total respiration rates and attempt to explain observed changes by describing how the microbial community jointly responds to the combined influence of protist predation and rising temperatures. We do so by incubating a microbial community from a local ephemeral pond in the presence and absence of two cosmopolitan ciliate protists of different size and at different temperatures. We ask (1) how does temperature influence microbial community biomass, structure, and function?, (2) how does protist presence influence these temperature responses?, and (3) are there direct temperature responses of the protists that in turn influence the microbial community?

We hypothesize that microbial biomass and respiration rate should increase with temperature, although we expect that effect to plateau over time (Yergeau et al., 2012; Bradford, 2013; Crowther and Bradford, 2013; Ye et al., 2020). We expect protist predation to decrease overall microbial biomass (Hahn and Höfle, 2001; Glücksman et al., 2010), which could reduce total respiration rates. We also hypothesize that predation effects should be dependent on protist size, as feeding rates are well known to increase with body size, while carrying capacity is known to decrease with predator body size (Englund et al., 2011; Rall et al., 2012; Zaoli et al., 2019). While little is known about the exact diet of these protists (but see recent unpublished work; Khadempour et al., 2021), we expect them to differ, at least minimally, because of gape-limitation (Glücksman et al., 2010): larger protists should be able to consume the same species that smaller protists do, plus some biofilm or colony-forming microbial taxa (Jürgens and Matz, 2002; Matz and Kjelleberg, 2005). Consequently, we hypothesize that differential consumption of microbial species by different protist species should result in changes in microbial composition (Hahn and Höfle, 2001; Glücksman et al., 2010) that may lead to changes in community structure and function. Last, we predict that protists themselves may respond to the imposed treatments by decreasing body size with temperature (temperature-size rule; Atkinson, 1994; Atkinson et al., 2006). These changes may in turn influence how they interact with the microbial community.

## Materials and Methods

### Water Collection, Microcosm Setup, and Incubation

We obtained an intact microbial freshwater community by collecting 40 l of surface water from a freshwater pond at Duke Forest (Gate 9, 36.019139, -78.987698, Durham, NC). To isolate the aquatic microbial community used in our experiment and remove larger protists, we filtered the entire water sample through autoclaved filters (11 μm pore size, Whatman) to remove debris, metazoans, and larger protists; then, we filtered through sterile GF/A filters (1.6 μm pore size, Whatman) to remove larger flagellates and fungal spores (Geisen et al., 2021). Bacteria and Archaea were retained in the filtrate. We also likely retained very small Eukaryotes like heterotrophic nanoflagellates. Removal of larger protists and metazoans from the source pond community was confirmed by visual inspection using a stereomicroscope (Leica M205C), then re-confirmed on control microcosms at the end of the experiment using fluid imaging (detailed below). We acknowledge that heterotrophic nanoflagellates are microbial predators (Pernthaler, 2005; Moreno et al., 2010), as are some members of the bacterial microbiome, but their presence was controlled across treatments by evenly dividing the filtered microbiome across samples (confirmed with the sequenced unincubated control samples). The resultant community was homogenized and incubated in 250 ml acid-washed and autoclaved borosilicate jars filled with a mixture of pond water filtrate (⅔, or 133 ml) and Carolina Biological Protist culture medium (⅓, or 67 ml). Following standard protist culturing procedures, we added two wheat seeds (~35 mg ea.) to serve as a carbon source and prevent issues of resource limitation (Altermatt et al., 2015). These wheat seeds were pre-autoclaved, and all sourced from the same batch, to control for any additional wheat seed-associated microorganisms. In total, our experiment comprised 120 microcosms, with ten replicates in each of six treatments, where half of each treatment was harvested at Day 12, and the other half at Day 24. Additionally, we harvested samples (*n* = 20) with just the pond microbiome, just before protist addition and temperature implementation Finally, we have four control samples (*n* = 1 ea.): Colpidium media, Tetrahymena media, pond microbiome, and a negative control (*n* = 1), containing the same volume of sterile protist media, to confirm axenic conditions throughout the incubation and subsequent processing. All microcosms were incubated under controlled environmental conditions in Percival AL-22 L2 growth chambers (Percival Scientific, Perry, Iowa) at 22°C, 10% light intensity (1700 lux), 75% humidity, and a 16:8 h day-night cycle (day length at time of collection). After a seven-day-pre-incubation period to allow incubation acclimation of the microbial communities, we harvested 20 microcosms as positive controls to assess the effects of incubation on bacterial community composition, relative to the original pond community. We acknowledge that laboratory incubations substantially impact microbial community structure and function, especially since most microorganisms are still unculturable (Steen et al., 2019). Negative controls of sterile media and water-only were extracted for genomic DNA alongside these 20 samples (*detailed below*).

### Experimental Treatments

After the initial seven-day-incubation period, microcosms were randomly assigned to treatments in a fully factorial experimental design with two levels of temperature (22°C, i.e., the water temperature on the day of collection or 25°C, a warming scenario simulating a + 3°C increase in temperature predicted by the IPCC report), and three levels of protist predation (no protists, presence of *Tetrahymena pyriformis* or presence of *Colpidium* sp.). We used *Tetrahymena pyriformis* (hereafter *Tetrahymena*) and *Colpidium sp.* (hereafter *Colpidium*), due to their putative generalist bacterivore habits (Foissner and Berger, 1996), their cosmopolitan distribution (Elliott, 1970), and, hence, their likelihood of playing a pivotal role in mediating the temperature response of microbial communities worldwide. Also, these protists have a large size difference (20–70 μm for *Tetrahymena* vs. 60–120 μm of *Colpidium*) which theory predicts should lead to differences in feeding and interaction strengths with their bacterial prey (Englund et al., 2011; Rall et al., 2012; Zaoli et al., 2019). These protist species originated from Carolina Biological Supply the culture collection and were cultured in the laboratory for 6 months prior to this experiment (Altermatt et al., 2015).

Protists were introduced by pipetting 0.5 ml of well-mixed protist stock cultures at carrying capacity into experimental microcosms. To control for the introduction of the microbes already occurring in the protist culture medium, we also added the same volume (1 ml) of a filtered and homogenized protist stock media, filtered of *Tetrahymena* and *Colpidium* cells, into all microcosms. The microcosms were thus assigned to one of 6 possible treatments: (1) 22°C, no protists; (2) 25°C, no protists; (3) 22°C, *Tetrahymena*; (4) 25°C, *Tetrahymena*; (5) 22°C, *Colpidium*; and (6) 25°C, *Colpidium*. Half of the microcosms in each treatment were harvested at Day 12 and the remaining half at Day 24 to assess whether observed responses changed over time in systematic ways.

### Community Biomass and Respiration Rate

We quantified total microbial biomass, microbial diversity, and total community respiration rate to assess the joint impacts of temperature, time, and protist predation on microbial community structure and function. As a proxy for total biomass, we measured the optical density at 600 nm wavelength (or OD600; Beal et al., 2020) of each microcosm (1/3 dilutions), using a Jenway 3,505 UV Spectrophotometer (Cole-Parmer, Vernon-Hills, IL, United States). Larger OD600 values (higher absorbance+scattering) indicate higher total biomass (Beal et al., 2020). Protists can also scatter light, albeit at a lower rate due to their larger size and lower densities (Thakur et al., 2021). Their presence should therefore increase OD600, all else being equal. If OD600 decreases in microcosms with protists relative to those without protists that would thus indicate a reduction in total bacterial biomass in the presence of protists (e.g., through protist predation).

We determined total community respiration rate using an optode-based real time OXY-4 SMA respirometer (PreSens, Regensburg, Germany; DeLong and Vasseur, 2012; Altermatt et al., 2015). Respiration rate was measured on 22 ml subsamples for 30 min, after a 30-min acclimation period, on a subset of all microcosms (*n* = 72). This was done at their original experimental temperature and the cross-treatment temperature (*n* = 36 microcosms, with two measurements each) to disentangle long term temperature effects from short-term impacts. For each microcosm, respiration rate was quantified twice, once at the temperature treatment at which it had been cultivated, and once at the other temperature treatment to control for possible acute effects of temperature on respiration rates. We therefore tested how all imposed treatments (Temperature, Time, and Protists) influenced respiration rates but also considered the temperature at which the respiration rate was measured. Respiration rates were estimated as the rate of change (slope) of the estimated oxygen concentration over time (in μmol O2/min; Supplementary Figures S1, S2; Appendix 1), following standard optode-based closed-system respirometry procedures (Tomlinson et al., 2018). Respiration rates did not differ significantly between the two temperatures at which they were measured (effect = −0.01 ± 0.12SE, *p* = 0.96), so readings for both temperatures from a single microcosm were averaged for subsequent analyses. To assess whether total community biomass or respiration rate changed with experimental treatment, we used linear models with protist presence (no protist, *Tetrahymena* or *Colpidium*), time (12 or 24 days), and temperature (22 or 25°C) as explanatory variables (and their possible interactions), and either biomass or respiration rates as response variables in R v4.0.2; R Core Team (2021), where the temperature, time, and protist treatments were all considered discrete variables. The input data were checked for clear departures from normality and homoscedasticity, but none were found. All measures of biomass, respiration rate, and bacterial community structure were measured at days 12 and 24.

### Protist Abundances and Traits

To disentangle potential effects of protist presence and abundance on microbial communities, we estimated protist population sizes through fluid imaging of 3 ml subsamples out of four microcosms from each treatment using a FlowCam (Fluid Imaging Technologies, Scarborough, ME, United States). Fluid imaging also yields high-resolution measurements of multiple cell traits like cellular volume and shape, and optical properties of the cells (Gibert et al., 2017; Wieczynski et al., 2021). We used these trait data to assess potential responses of the protists to imposed experimental conditions as well as potential responses of the microbial communities to both protist traits and densities (*detailed below*). We focused on nine different phenotypic characteristics: five measurements of shape and size (length, area, volume, circularity, and aspect ratio) and four measurements of optical properties of the cells (sigma-intensity and three components of hue: Red/Green, Red/Blue, and Blue/Green ratios). These measurements were taken on days 12 and 24. We assessed whether protist population counts changed with either temperature or time using linear models, and we assessed whether protist phenotypes responded over time and with temperature using perMANOVA (Anderson, 2001, 2017) using the *vegan* package in R (v2.5.6; Oksanen et al., 2019). The input data were checked for clear departures from normality and homoscedasticity, but none were found.

### Amplicon Sequence Data Processing and Bacterial Community Structure Analysis

We used 16S rRNA amplicon sequencing to examine the impacts of temperature, time, and protists on microbial community structure. After the incubation period (12 or 24 days), we collected the microbial communities by filtering 200 ml from each microcosm into gamma-irradiated 0.2 μm nitrocellulose membranes (Advantec, Taipei, Taiwan) and stored the filters at -20°C until DNA extraction. Total genomic DNA was extracted from each filter using DNeasy PowerWater DNA Extraction Kits (Qiagen, Hilden, Germany), modified with a heating step (60°C) before the initial vortexing step to maximize lysis across different microbial cell types. We fluorometrically quantified the genomic DNA concentrations with Qubit (Thermo Fisher, Waltham, MA, United States) and sent an equimolar set of genomic DNA samples to the Research Technology Support Facility (RTSF) at Michigan State University for amplicon prep and sequencing. We targeted the V4 hypervariable region of the 16S rRNA gene using the standard 515F/806R universal primers with 12 bp Golay barcodes (Caporaso et al., 2011). RTSF sequenced our samples with Illumina MiSeq (PE 250 bp, V2 chemistry) and returned 9,069,268 raw reads (average/sample: 62,981 reads), publicly available at EMBL-ENA with project accession numbers available upon request, and listed here upon publication.

We processed the raw fastq sequence data through Dada2 (v1.16.0; Callahan et al., 2016) in R (v4.0.2; R Core Team, 2021), to trim and filter low quality sequence reads [full-length reads (250 bp) retained without ambiguous bases (maxN = 0), relaxed expected error in the reverse reads (maxEE = (2,5)); default phiX removal and truncation of reads where quality score drops below 2] and calculate error rates for denoising and merging the pair-ends into 3,801 non-chimeric representative amplicon sequence variants, or ASVs. These representative ASVs were further curated with Lulu to reduce artificially inflated diversity due to amplification and sequencing errors, resulting in 1423 representative ASVs (Frøslev et al., 2017).

We taxonomically identified chloroplast and mitochondrial 16S rRNA sequences using the Silva 138 reference taxonomy (Quast et al., 2013), removing 315 ASVs for a final 16S rRNA representative set of 1,108 ASVs. This final representative ASV set was aligned to the Silva 138 NR full-length 16S rRNA alignment with MAFFT (Katoh and Frith, 2012) using default settings, and subsequently trimmed and masked to the V4 region. We then updated the trimmed V4 alignment to estimate a phylogeny using the iterative algorithm of PASTA (Mirarab et al., 2015) and used this final set of ASVs to update the corresponding sample ASV community table.

For alpha-diversity estimates, we rarefied all microbial 16S rRNA samples to a sequencing depth of 8,600 to maximize sampling depth while retaining the majority of samples. We treated the data as compositional for all other analyses by using a variance-stabilizing transformation (VST) of the ASV community table without singleton ASVs (Gloor et al., 2017), using DESeq2 (v1.12.3; McMurdie and Holmes, 2013). We calculated a comprehensive range of alpha-diversity indices, from observed ASV richness and Shannon-Weiner to Pielou’s evenness index and abundance-weighted phylogenetic diversity (weighted Unifrac) on each rarefied sample using “core-metrics-phylogenetic” in Qiime2 (v2020.8; Bolyen et al., 2019). We used ANOVA to test for individual and interactive effects of experimental treatments on bacterial alpha diversity.

To examine the impacts of temperature, incubation time, and protists on the overall structure of the bacterial communities, we used principal component analysis on an abundance-weighted bray-curtis distance matrix of the VST table in *vegan* R (v2.5-6; Oksanen et al., 2019). We tested for individual and interactive treatment effects with a perMANOVA using the adonis() function in *vegan* R (v2.5-6; Oksanen et al., 2019). We performed multi-level pairwise comparisons of the community data using the pairwise.adonis() function in the pairwiseAdonis R package (v0.0.1; Martinez Arbizu, 2017). We also examined differences among group community variation—or spread in ordination space—using the betadisper() function (PERMDISP2; Anderson et al., 2006) in *vegan* R (v2.5-6; Oksanen et al., 2019), which uses multivariate homogeneity of group dispersions, or the multivariate form of a Levene’s test (O’Neill and Mathews, 2000; Anderson, 2006).

For treatments imposing significant changes in bacterial community structure, we also identified potential positive or negatively responding bacterial taxa (ASVs) to increased temperature or to the presence of a protist predator. We employed two distinctive methods of indicator taxa analysis: categorical *indicspecies* in R (De Cáceres and Legendre, 2009) and a direct gradient analysis, threshold indicator taxa analysis (TITAN) in R (Baker and King, 2010). With indicspecies, we identified ASVs with significant differential abundance patterns by treatment level using multi-level pattern analysis with the multipatt() function (De Cáceres and Legendre, 2009). In contrast, with TITAN, we identified ASVs significantly associated with changes in measured protist phenotypic traits, specifically protist size, and protist abundance, by separately regressing each bacterial ASV against each protist trait. TITAN outputs provided pure and reliable positive and negative indicator ASVs (75% purity and reliability thresholds), as well as individual and community-level abundance thresholds, for each protist trait gradient. All data and code used for these analyses are available upon request, with github repo listed here upon publication.

## Results

### Temperature and Time Affect Microbial Biomass, Function, and Structure

**Figure 1:**
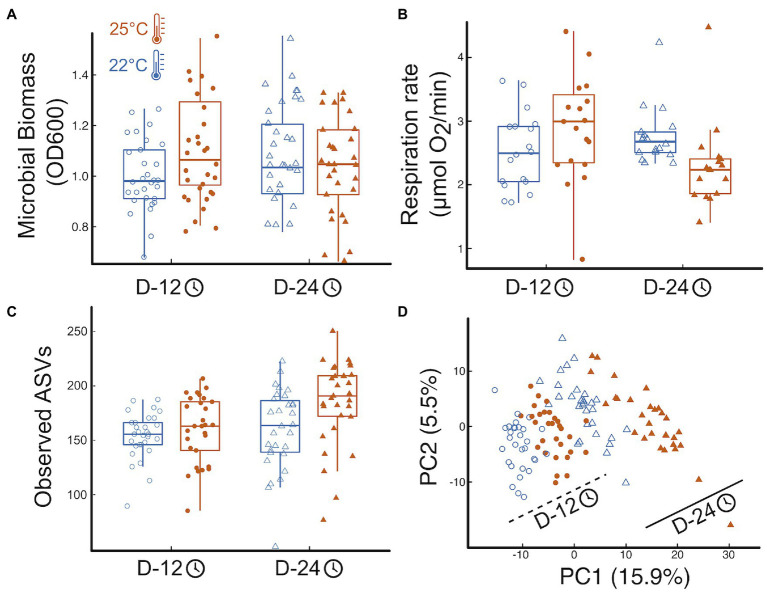
Temperature impacts on microbial function and community structure. **(A)** Boxplots of the treatment effects on total microbial biomass, **(B)** the effects on total microbial respiration, here as oxygen consumption, **(C)** the effects on microbial community richness, here observed ASVs (see Fig S5 for other diversity indices), and **(D)** the effects of temperature and time on the microbial community structure (microbial abundance inferred from sequence data). Shapes represent incubation time: Day12—circles, Day 24—triangles; temperature marked by point fill: 22°C—empty point, 25°C—color filled point. Panels include all data pooled across protist and no protist treatments.

Temperature and incubation time had interactive effects on total microbial biomass: warmer temperature resulted in larger OD600 but that effect disappeared over time (Figure 1A, effect_Temp_ = 0.08 ± 0.04SE, *p* = 0.08; effect_Time_ = 0.10 ± 0.04SE, *p* = 0.019; effect_Temp x Time_ = −0.14 ± 0.06SE, *p* = 0.02, more details in Appendix 2, where SE stands for the standard error of the mean). Respiration rate also showed significant interactive effects of time and temperature similar to those found for bacterial biomass: respiration increased with temperature at first but that effect was reversed over time (Figure 1B, effect_Temp_ = 0.62 ± 0.29SE, *p* = 0.036; effect_Time_ = 0.46 ± 0.20SE, *p* = 0.025; effect_Temp x Time_ = −0.90 ± 0.29SE, *p* = 0.003, Appendix 2).

Temperature and time, but not their interaction, influenced microbial community structure, with increased alpha-diversity and a significant change in the bacterial community composition, with elevated temperature and with time. (Figure 1C; Appendix 3). Mimicking biomass and respiration results, microbial community structure was significantly affected by temperature, time, and their interaction (Figure 1D). The microbial communities were primarily structured by incubation time, with 13.3% of the variation explained by community shifts from Day 12 to Day 24 harvest (*p = 0.001*). Temperature (22°C vs. 25°C) explained 5.5% of the community variation across all harvesting time points (*p = 0.001*, Figure 1D), while the interaction of temperature and time explained 3.2% of the variation in microbial community structure (*p = 0.001*, Figure 1D). Post-hoc tests revealed that all four treatment combinations resulted in significantly distinct groupings of microbial community structure (*p.adj = 0.006*). Finally, increased temperature and incubation time imposed significant increase to beta-dispersion (temp: *p.adj = 0.003*, time: *p.adj = 0.001*), with wider spread in group dispersion at the warmer temperature in the early harvested microbiomes (*p.adj = 0.045*; Figure 1D).

### Effects of Protist Predation on Microbial Function and Community Structure

**Figure 2:**
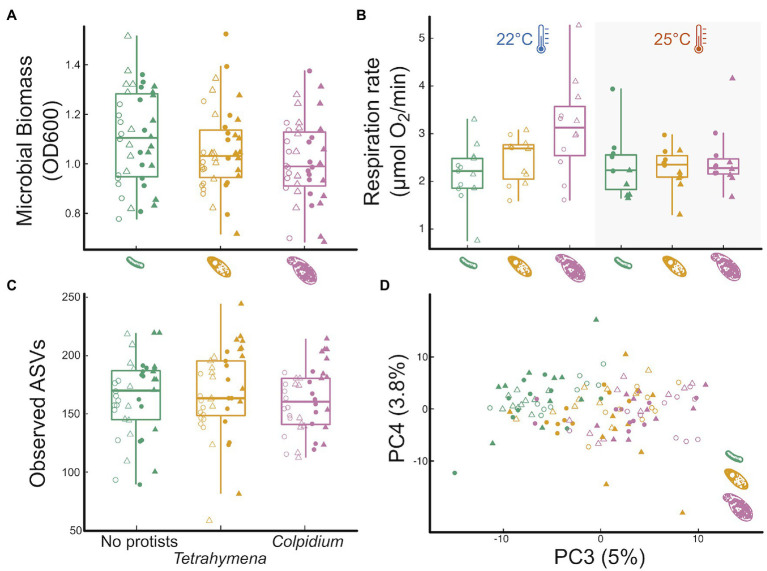
Influence of protist presence and species on pond microbial community function and community structure. The effect of the absence of protists (green), *Tetrahymena* (yellow) or *Colpidium* (fuchsia) on: **(A)** optical density (600 nm) as a proxy for total microbial biomass, **(B)** total microbial respiration (O2 consumption rate), **(C)** microbial community richness (observed ASVs; see Supplementary Figure S6 for other diversity indices), and **(D)** a principal component analysis of microbial community structure. Shapes represent incubation time: Day12—circles, Day 24—triangles; temperature marked by point fill: 22°C—empty point, 25°C—color filled point.

The larger protist (*Colpidium*) significantly reduced OD600 biomass relative to the no protist treatment, while the smaller *Tetrahymena* did not (Figure 2A, effect_Colp_ = −0.09 ± 0.04SE, *p* = 0.015; effect_Tetra_ = −0.05 ± 0.04SE, *p* = 0.19). Protist effects on OD600 biomass did not interact with time or temperature (Supplementary Table S1; Supplementary Figure S3; Appendix 2). *Tetrahymena* had no noticeable effect on microbial respiration rate either (Figure 2B, effect_Tetra_ = −0.05 ± 0.04SE, *p* = 0.19). However, *Colpidium* had a significant effect on respiration that interacted with temperature: in the presence of *Colpidium,* total respiration was lower at 25°C than at 22°C (effect_Colp_ = 0.99 ± 0.25SE, *p* < 0.01; effect_Colp x Temp_ = −0.87 ± 0.36SE, *p* = 0.017; Figure 2B).

Predator presence had no significant effects on any of the measured alpha-diversity indices of the microbial communities (ASV richness, *p = 0.6*; phylogenetic diversity, *p = 0.8*; Shannon-Wiener diversity, *p = 0.5*; or community evenness, *p = 0.4*, Figure 2C; Appendix 3). However, both protists significantly affected microbial community composition (Figure 2D), although neither one interacted with temperature or time (protist and temperature interaction: *p* = 0.22; protist and time interaction: *p* = 0.25; and protist, time, and temperature interaction: *p* = 0.60). Protist treatments explained 6.7% of the variation in microbial community structure (*p = 0.001*). Microbial communities exposed to either protist species significantly differed from the no protist microbial communities (*Tetrahymena*: *p.adj = 0.001*; *Colpidium*: *p.adj = 0.001*) and also differed between the two protist species treatments (*p.adj = 0.004*). Group dispersion analysis of beta diversity revealed no differences in the degree of group variation in community structure among protist treatment levels (*p.adj = 0.239*).

### Feedbacks on Protist Abundance and Traits

**Figure 3:**
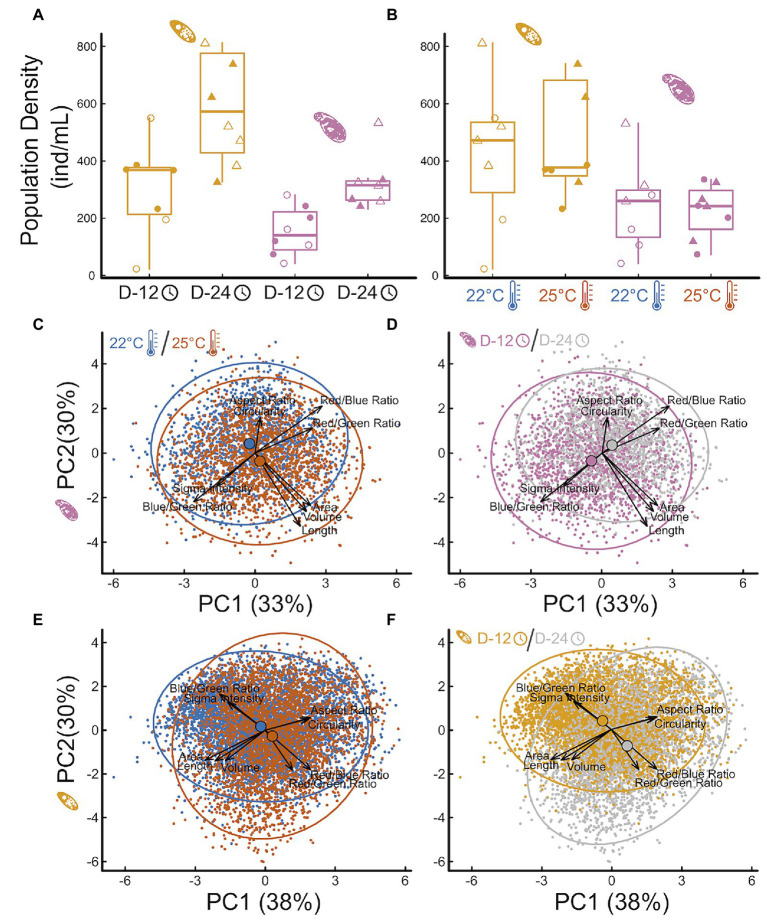
Impact of temperature treatments and incubation time on protist density and morphology. The effect of incubation time **(A)** and temperature treatment **(B)** on *Tetrahymena* (yellow) and *Colpidium* (pink) density (cells/mL) in the microcosms, and principal component analysis of multivariate protist phenotypes **(C–F)**: *Colpidium* **(C,D)** and *Tetrahymena* **(E,F)**, colored by temperature **(C,E)** and by time **(D,F)**. Vectors represent the principal components loadings of each measured phenotypic characteristic, including shape, size, optical depth, and hue.

Protist abundance increased over time (Figure 3A effect_Colp_ = 172.05 ± 46.77SE, *p* = 0.002; effect_Tetra_ = 344.9 ± 132.9SE, *p* = 0.02), as expected, but final abundance was independent from temperature (Figure 3B effect_Colp_ = −16.43 ± 66.66SE, *p* = 0.89; effect_Tetra_ = 120.9 ± 160.3SE, *p* = 0.46). This indicates that temperature effects on protist abundances are unlikely to explain, alone, observed effects on microbial communities and respiration rate. Both time and temperature influenced protist traits independently and interactively (Figures 3C–F; Supplementary Tables S4**–**S7; Appendix 4), but each species responded in slightly different ways. Contrary to the temperature-size rule, *Colpidium* sp. responded to increasing temperatures by becoming larger and more elongated (Figure 3C, perMANOVA *p* < 0.01, Supplementary Tables S4, S5; Appendix 4). On the other hand, *Tetrahymena* response was consistent with a temperature-size rule, becoming smaller and rounder with temperature (Figure 3E, perMANOVA *p* < 0.01, Supplementary Tables S6, S7; Appendix 4). Over time, however, *Colpidium* got smaller and shorter (Figure 3D, perMANOVA *p* < 0.01, Supplementary Tables S4, S5; Appendix 4), while temperature effects on *Tetrahymena* were exacerbated over time (Figure 3F, perMANOVA *p* < 0.01, Supplementary Tables S6, S7; Appendix 4). Both protists showed changes in optical properties as time went by Figures 3D,E, perMANOVA *p* < 0.01, suggestive of changes in cellular contents.

### Density and Trait Effects of Protists on Taxa-Specific Responses

**Figure 4:**
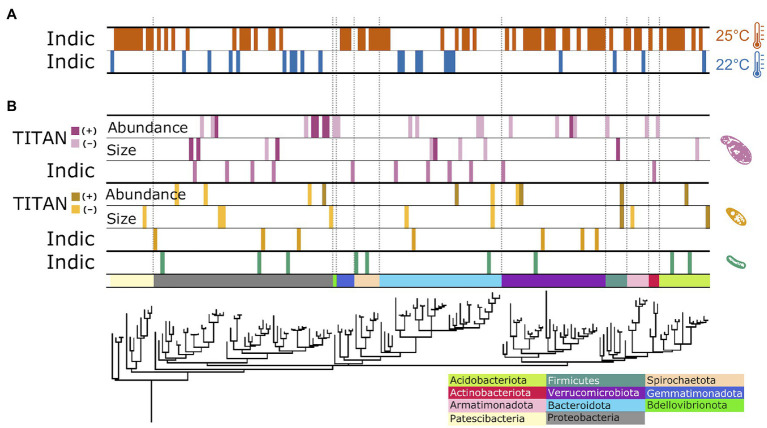
Phylogenetic distribution of treatment impacts on individual bacterial ASVs. Responder ASVs showing consistent change in relative abundance to temperature **(A)** and protist **(B)** treatments. Data strips labeled, “Indic,” represent ASVs positively correlated with a particular temperature treatment level, generated from multi-level pattern analysis output; data strips labeled, “TITAN,” show responding ASVs to changes in specific protist species’ traits, analyzed with direct gradient analysis.

The observed changes in overall bacterial community structure were likely driven by the 113 ASVs that exhibited significant changes in relative abundance (8% of 1,423 total ASVs) to the imposed experimental treatments (Figure 4; Appendix 5). Of these responders, 91 ASVs responded significantly to temperature, with 76.9% positively responding to increased temperature and 23.1% showing decreased relative abundance with elevated treatment (Figure 4A). The ASVs flourishing under elevated temperature were largely clustered into several phyla: Verrucomicrobia (12 ASVs), Proteobacteria (10 ASVs), the basal Patescibacteria (9 ASVs), Bacteroidota (7 ASVs), and Spirochaetota (7 ASVs); while the other 25 “warm responders” were spread across ten additional bacterial phyla. In contrast, ASVs thriving under ambient temperatures (21 ASVs) were distributed across the entire bacterial phylogeny (Figure 4A).

Of the 113 responding ASVs, 23.9% (27 ASVs) exhibited significant shifts in relative abundance to the presence, or absence, of the predator protists (Figure 4B). The presence of *Colpidium* resulted in more responding ASVs (11 ASVs) compared to *Tetrahymena* (7 ASVs) or the no protist treatment (9 ASVs). Responders were distributed throughout the bacterial domain. With TITAN analysis, we also identified 47 ASVs as indicators to gradients of protist cell density and body size (Figure 4B). Seven indicators were negatively associated with *Tetrahymena* body size, while two ASVs positively responded to increased cell size. The density of *Tetrahymena* density corresponded to five positive and five negative responders, distributed across five phyla. Two ASVs responded consistently to cell density and size: a negative indicator ASV_45, identified as *Paenibacillus* spp. (Chitinophagaceae), and a positive responder, ASV_269 in Selenomonadaceae. *Colpidium* cell density and body size resulted in 82% more responding ASVs than to *Tetrahymena* traits. Most of the responders were impacted by *Colpidium* density (21 ASVs), of which the bulk (15 ASVs) responded negatively to more *Colpidium* cells. No ASVs responded to both Colpidium density and cell size, but one responder (ASV 78), in the genus *Afipia*, positively responded to the density of both predators (Figure 4B; Supplementary Table S10; Appendix 5). ASVs from Proteobacteria, Bacteroidota, and Verrucomocrobiota seemed to respond to both protists, while ASVs from Gemmatimonadota and Actinobacteria only responded to Colpidium (Figure 4B), thus suggesting some level of specificity to predation by protists, but also to protist species.

## Discussion

Microbes strongly influence the global carbon cycle through respiration and assimilation of both labile and recalcitrant forms of carbon (Jackson et al., 2017). Understanding how changes in environmental temperature may influence microbiome function in general, and respiration rates, in particular, are crucial to hone our ability to forecast future warming trends (Jansson and Hofmockel, 2020). Our study shows how temperature determines both bacterial community structure and overall microbial respiration rate in a temperate pond microbial community (Figure 1). We also show how predation by one of the most important bacterivores worldwide, protist ciliates (Gao et al., 2019; Oliverio et al., 2020), mediates temperature effects on respiration (Figures 2–4), through phylum-specific bacterial responses to protist density and size (Figure 4).

Our results show that temperature directly influences community function, owing, in part, to shifts in community structure over time and across temperatures (Figure 1). However, the effects of temperature on function were reversed over time (Figure 1C), which is consistent with other studies showing that while total microbial respiration increases with temperature at first, that effect is short lived or even fully reversed as time elapses (Yergeau et al., 2012; Bradford, 2013; Crowther and Bradford, 2013; Ye et al., 2020). To explain these changes, previous studies have invoked shifts in carbon use efficiency (Frey et al., 2013). While we cannot rule out the possibility of decreased availability of labile carbon within our microcosms, we have attempted to control for that by adding wheat seeds, which provided a slow release of labile carbon to the incubated communities over time (Altermatt et al., 2015). On the other hand, both temperature and time led to large shifts and increased variability in community structure (Figure 1D), with the warmer temperature leading to a higher relative abundance of some bacterial taxa over others (Figure 4A; Martiny et al., 2013; Isobe et al., 2020). These results thus suggest a possible causal relationship between changes in community structure and function, as proposed by others (Hall et al., 2018), and despite such changes being rarely observed (Rocca et al., 2015; Graham et al., 2016; Fang et al., 2020). While changes in carbon use efficiency have been recently accounted for in state-of-the-art forecasting models (Allison et al., 2010; Sinsabaugh et al., 2013), changes in bacterial community structure have not Sulman et al. (2018). Our results show one interesting way in which changes in environmental conditions may influence bacterial community structure and function.

Secondly, we show that predation by larger protists, like *Colpidium* sp., influences the impacts of temperature on microbial community function (Figures 2A,B). Indeed, protist predation resulted in changes in total biomass (Figure 2A) and microbial respiration (Figure 2B), that interacted with temperature. Predation by the larger *Colpidium sp.* actually led to a reversal of the temperature effect on respiration rate (Figure 2B), which may be partly due to a larger feeding rate but also to differences in carrying capacity. Indeed, carrying capacity is well known to decline with body size with a slope of -¾ (in double log) and is one of the ways in which predator body size can influence the biomass and composition of the resource community (Englund et al., 2011; Rall et al., 2012; Zaoli et al., 2019). Interestingly, a recent paper showed that soil decomposition rates increased in the presence of the protist *Physarum polycephalum* at low temperatures but that effect disappeared at a warmer temperature (Geisen et al., 2021). Our results confirm—and extend—those of Geisen and others to a different protist system, and to a freshwater microbial community; thus, suggesting this might be a more general pattern than expected. If further confirmed, this may also indicate that predation by protists could reduce total microbial respiration in warmer climates, thus representing a poorly understood but potentially important biotic control on the global carbon cycle.

Thirdly, protist effects on total microbial biomass (Figure 2A) and respiration rate (Figure 2B) were, as hypothesized, size-dependent (Figures 3A,B, 4B). Further analysis also revealed that these effects on bacterial community structure were likely due to individual bacterial taxa differentially responding to protist density and size (Figure 4B), thus suggesting that strong size-dependencies may be at play. Previous studies have shown that protist predation can select for bacterial size (Hahn and Höfle, 2001; Pernthaler, 2005; Erktan et al., 2020), and both negative and positive associations with different protists have also been shown (Oliverio et al., 2020). In addition to the direct predator effects on responding bacterial taxa, we cannot rule out indirect effects of the predator presence—due to higher order interactions—to explain the abundance shifts of certain responding bacteria (Karakoç et al., 2018; Mickalide and Kuehn, 2019). Our results add to this growing literature by linking changes in protist density and size to specific bacterial responders (Figure 4B). Lastly, our results showed that the traits of both protist predators also responded to imposed experimental conditions, albeit in different ways. While size is well known to influence consumer-resource interactions (Gilbert et al., 2014; DeLong et al., 2015; Gauzens et al., 2020), protist size and shape can and often do respond to foraging (Atkinson et al., 2006; DeLong et al., 2014; Gibert et al., 2017; Tan et al., 2021), thus potentially resulting in a feedback between predator and prey phenotypes. Since the microbial communities themselves changed in structure with time and temperature, this study cannot tease apart the direct effects of temperature on protist responses from those mediated by microbial community temperature responses. However, we do show they both occur in tandem, thus underscoring the importance of understanding how these reciprocal effects may lead to shifts in the function of microbial communities as temperatures increase.

While our results uncovered interesting patterns about predation influence on the temperature response of microbial communities, there also are shortcomings to our findings that need to be accounted for to fully understand the full scope of these results. For example, the incubation of the microbial communities led to significant departures from the initial pond composition, which are more substantial than the subsequent community shifts observed in the experiment (Supplementary Figure S7), which is also expected because most microorganisms are still unculturable (Steen et al., 2019). One possibility is that our microcosms were artificially awash with nutrients, thus selecting for a microbial composition that would not naturally prevail in more oligotrophic pond conditions. To better understand how common the results shown here may actually be, these abiotic and biotic treatments should also be studied in mesocosms or other semi-natural experimental settings. Another potential contributor is the presence of heterotrophic nanoflagellates, which were likely retained in our intact community because of their very small size. Despite the presence of these microbial predators, which were controlled across all treatments, the presence of the larger protists still imposed effects on the bacterial community structure and total respiration of our incubation. Our experimental temperature treatments also did not account for diurnal and seasonal temperature fluctuations, nor could they inform us about any broader effects of seasonal changes in temperature. Seasonality, in particular, is likely also shifting as temperatures rise globally (Easterling et al., 2000; Meehl and Tebaldi, 2004; Rahmstorf and Coumou, 2011; Rummukainen, 2012), but whether those effects differ from those of changes in mean temperatures, as shown here, are unknown. Last, the short-term nature of our experimental manipulations necessarily reduces the possible scope of our inference, even though both the nature and magnitude of the effects reported here seem large enough to be of importance beyond our specific set up. Last, predator–prey interactions are well known to oscillate under some conditions (Grunert et al., 2021), so it is also possible that natural predator–prey oscillations may be partly responsible for observed changes in community structure and abundance over time.

Our results emphasize the dynamic nature of temperature effects on microbial community structure and function as well as how a neglected biological factor (protist predation) influences such responses. We show how protist predation can mediate temperature effects on microbial communities, how such impacts are dependent on the body size and density of the predator, and how microbial responses to temperature may in turn influence the traits of these microbial consumers. Our study suggests interesting mechanisms through which microbial predation may influence the global carbon cycle.

## Data Availability Statement

The datasets presented in this study can be found in online repositories. The names of the repository/repositories and accession number(s) can be found in the article/Supplementary Material.

## Author Contributions

JDR and JPG conceived the study and wrote the manuscript with significant input from all other authors. JDR, AY, and JPG designed and conducted the experiments. JPG, AY, and MS processed and analyzed the microbial biomass, respiration, and protist trait data. JDR and MS processed and analyzed the bacterial community data. All authors contributed to the article and approved the submitted version.

## Funding

This work was supported by JPG’s Duke University startup funds and a U.S. Department of Energy, Office of Science, Office of Biological and Environmental Research, Genomic Science Program under Award Number DE-SC0020362.

## Conflict of Interest

The authors declare that the research was conducted in the absence of any commercial or financial relationships that could be construed as a potential conflict of interest.

## Publisher’s Note

All claims expressed in this article are solely those of the authors and do not necessarily represent those of their affiliated organizations, or those of the publisher, the editors and the reviewers. Any product that may be evaluated in this article, or claim that may be made by its manufacturer, is not guaranteed or endorsed by the publisher.
